# Antimicrobial Susceptibility Survey of Invasive Haemophilus influenzae in the United States in 2016

**DOI:** 10.1128/spectrum.02579-21

**Published:** 2022-05-10

**Authors:** Caelin C. Potts, Lorraine D. Rodriguez-Rivera, Adam C. Retchless, Sean A. Buono, Alexander T. Chen, Henju Marjuki, Amy E. Blain, Xin Wang

**Affiliations:** a Division of Bacterial Diseases, National Center for Immunization and Respiratory Diseases, Centers for Disease Control and Prevention, Atlanta, Georgia, USA; b Weems Design Studio, Inc., Division of Bacterial Diseases, National Center for Immunization and Respiratory Diseases, Centers for Disease Control and Prevention, Atlanta, Georgia, USA; c IHRC, Inc., Division of Bacterial Diseases, National Center for Immunization and Respiratory Diseases, Centers for Disease Control and Prevention, Atlanta, Georgia, USA; d Division of Bacterial Diseases, National Center for Immunization and Respiratory Diseases, Centers for Disease Control and Prevention, Atlanta, Georgia, USA; Brown University

**Keywords:** *Haemophilus influenzae*, antimicrobial susceptibility, invasive disease

## Abstract

Antibiotics are important for the treatment and prevention of invasive Haemophilus influenzae disease. Reduced susceptibility to clinically relevant drugs, except ampicillin, has been uncommon in the United States. Susceptibility of 700 invasive H. influenzae isolates, collected through population-based surveillance during 2016, was assessed for 15 antibiotics using broth microdilution, according to the CLSI guidelines; a subset of 104 isolates were also assessed for rifampin susceptibility using Etest. Genomes were sequenced to identify genes and mutations known to be associated with reduced susceptibility to clinically relevant drugs. A total of 508 (72.6%) had reduced susceptibility to at least one antibiotic and more than half of the isolates exhibited reduced susceptibility to only one (33.6%) or two (21.6%) antibiotic classes. All tested isolates were susceptible to rifampin, a chemoprophylaxis agent, and <1% (*n* = 3) of isolates had reduced susceptibility to third generation cephalosporins, which are recommended for invasive disease treatment. In contrast, ampicillin resistance was more common (28.1%) and predominantly associated with the detection of a β-lactamase gene; 26.2% of isolates in the collection contained either a TEM-1 or ROB-1 β-lactamase gene, including 88.8% of ampicillin-resistant isolates. β-lactamase negative ampicillin-resistant (BLNAR) isolates were less common and associated with *ftsI* mutations; resistance to amoxicillin-clavulanate was detected in <2% (*n* = 13) of isolates. The proportion of reduced susceptibility observed was higher among nontypeable H. influenzae and serotype e than other serotypes. US invasive H. influenzae isolates remain predominantly susceptible to clinically relevant antibiotics except ampicillin, and BLNAR isolates remain uncommon.

**IMPORTANCE** Antibiotics play an important role for the treatment and prevention of invasive Haemophilus influenzae disease. Antimicrobial resistance survey of invasive H. influenzae isolates collected in 2016 showed that the US H. influenzae population remained susceptible to clinically relevant antibiotics, except for ampicillin. Detection of approximately a quarter ampicillin-resistant and β-lactamase containing strains demonstrates that resistance mechanisms can be acquired and sustained within the H. influenzae population, highlighting the continued importance of antimicrobial resistance surveillance for H. influenzae to monitor susceptibility trends and mechanisms of resistance.

## INTRODUCTION

Haemophilus influenzae is a Gram-negative coccobacillus that can cause life-threatening infections, including bacteremic pneumonia, bacteremia without focus, and meningitis (1–3). Vaccination efforts have resulted in a dramatic reduction in the burden of invasive disease caused by H. influenzae serotype b (Hib) (1, 4); however, other serotypes (Hia, Hic, Hid, Hie, and Hif) as well as unencapsulated, nontypeable H. influenzae (NTHi) are not vaccine-preventable and continue to cause invasive disease in the United States (4). Among all age groups, the national annual incidence for invasive H. influenzae disease was 1.7 cases per 100,000 population during 2009–2015, with a case fatality of 14.5% (4). In addition to invasive diseases, H. influenzae can also cause more mild infections, including conjunctivitis, sinusitis, and acute otitis media (2, 5).

The severity and site of H. influenzae infection affect the recommendations for antibiotic treatment and chemoprophylaxis (2). Meningitis is treated with third generation cephalosporins, specifically ceftriaxone and cefotaxime. Although not recommended for invasive infections or disease in immunocompromised individuals, ampicillin may also be used for treatment after the H. influenzae strain is confirmed to be susceptible (2). In addition, acute otitis media in children is commonly treated with amoxicillin; if treatment failure is observed, amoxicillin-clavulanate, cefdinir, cefpodoxime, cefuroxime or ceftriaxone can be used (2). For chemoprophylaxis, rifampin is recommended for household and/or daycare contacts of individuals with invasive Hib or Hia disease, based on criteria, including age and vaccination status of the contacts (2).

Antimicrobial susceptibility of bacterial pathogens, including H. influenzae can be assessed by various methods (disk diffusion, agar dilution, broth microdilution, or gradient strip diffusion method [Etest]) according to international standards provided by the Clinical and Laboratory Standards Institute (CLSI) and the European Committee on Antimicrobial Susceptibility Testing (EUCAST) (6). Agar dilution or broth microdilution is considered the gold standard that accurately measure the MIC. However, this method is expensive and technically complex, requiring robust quality control/quality assurance testing procedures. Etest incorporates the principle of disk diffusion and agar dilution tests and uses a predefined antibiotic gradient to determine the MIC in micrograms per milliliter (μg/mL). Etest can rapidly test the anaerobes and fastidious bacterial species for antimicrobial susceptibility, including H. influenzae (7). In general, pairwise comparison of disk diffusion, agar dilution, broth microdilution, and Etest showed very good categorical agreement (>88%) (8); however, some predominantly minor categorical discrepancies were observed (8). While broth microdilution remains a reference method for H. influenzae antimicrobial susceptibility evaluation, one study suggested this method is not able to effectively detect heteroreistance in H. influenzae, compared to Etest and agar dilution, which may have caused an underestimate of the extent of heteroresistance in this organism (7, 9).

Trends in US H. influenzae susceptibility were studied during the 1990s and early 2000s. Reduced susceptibility was most consistently observed for ampicillin, with approximately a quarter to a third of isolates exhibiting resistance (10–17); resistance was predominantly caused by β-lactamase expression (mostly TEM-1, but ROB-1 has also been detected) (5, 18, 19). β-lactamase negative ampicillin-resistant (BLNAR) isolates have been also characterized and are associated with mutations in *ftsI*, which encodes penicillin binding protein 3 (PBP3) (5, 20, 21); *ftsI* mutations that result in amino acid substitutions are categorized into group I (N526K), group II (R517H), group III (N526K, S385T, L389F), and group III-like (R517H, S385T, L389F) (20–23). Mutations in *ftsI* have also been implicated in reduced susceptibility to cephalosporins; however, a true causal relationship remains to be described (20, 22, 24, 25). In addition to the β-lactams, reduced susceptibility to trimethoprim-sulfamethoxazole and clarithromycin have also been commonly observed in previous susceptibility surveys, while isolates have been predominantly susceptible to the other antibiotics assessed, including amoxicillin-clavulanate, cefuroxime, ceftriaxone, rifampin, tetracycline, levofloxacin, and chloramphenicol (10–17).

Here, we determined the antimicrobial susceptibility among invasive H. influenzae isolates collected during 2016 through US population-based Active Bacterial Core surveillance. Isolates were assessed for susceptibility to 15 antibiotics by broth microdilution; susceptibility to a 16th antibiotic, rifampin, was also determined by Etest for a subset of isolates. The mechanisms of resistance were investigated using whole-genome sequencing.

## RESULTS

The invasive H. influenzae isolate collection (*n* = 700) consisted of 26.6% typeable (Hia *n* = 51, Hib *n* = 12, Hie *n* = 34, Hif *n* = 89) and 73.4% NTHi (*n* = 514) isolates. Altogether, the collection included 136 sequence types, which were associated with the isolate serotype (Table S1); 10 isolates could not have a sequence type assigned, due to a missing *fucK* gene, which is required for MLST analysis. A total of 192 isolates (27.4%) were susceptible to all antibiotics assessed, while the remaining 508 (72.6%) had reduced susceptibility to at least one antibiotic (Table 1). While more than half of the isolate collection exhibited reduced susceptibility to only one (33.6%) or two (21.6%) antibiotic classes, 122 (17.2%) had reduced susceptibility to three or more (Table 1).

**TABLE 1:** Reduced susceptibility^*a*^ by number of antibiotic classes among invasive H. influenzae isolates, collected through Active Bacterial Core Surveillance, United States, 2016 (n = 700^*b*^)

Antibiotic class combinations^*c*^ with reduced susceptibility	No. of isolates with reduced susceptibility
0 Classes, n (%)	192 (27.4)
No reduced susceptibility	192
1 Class, n (%)	235 (33.6)
Penicillins	33
Cephalosporins	7
Macrolides	125
Sulfonamides	70
2 Classes, n (%)	151 (21.6)
Penicillins cephalosporins	37
Penicillins carbapenems	1
Penicillins macrolides	36
Penicillins sulfonamides	4
Cephalosporins macrolides	8
Cephalosporins sulfonamides	5
Macrolides sulfonamides	58
Fluoroquinolones sulfonamides	2
3 Classes, n (%)	97 (13.9)
Penicillins cephalosporins carbapenems	1
Penicillins cephalosporins macrolides	50
Penicillins cephalosporins sulfonamides	28
Penicillins macrolides sulfonamides	1
Penicillins tetracyclins sulfonamides	2
Cephalosporins macrolides sulfonamides	13
Cephalosporins fluoroquinolones sulfonamides	1
4 Classes, n (%)	22 (3.1)
Penicillins cephalosporins macrolides sulfonamides	22
5 Classes, n (%)	2 (0.3)
Penicillins cephalosporins carbapenems macrolides sulfonamides	1
Penicillins cephalosporins macrolides tetracyclins sulfonamides	1
6 Classes, n (%)	1 (0.1)
Penicillins cephalosporins amphenicols macrolides tetracyclins Sulfonamides	1

a Reduced susceptibility is defined as any isolate that was intermediate, resistant or nonsusceptible.

b Invasive isolates were collected through the Active Bacterial Core Surveillance program, which is active, population- and laboratory-based surveillance and includes 10 catchment areas that covered approximately 44.2 million US residents (13.7% of the population in 2016) (32). During 2016, 767 of 866 confirmed cases (88.6%) included in the ABCs had isolates submitted to CDC; among those, 700 were randomly selected for broth microdilution testing. A subset of the 700 isolates (*n* = 104) were also assessed for rifampin susceptibility by Etest.

c Antibiotic classes were defined as the following: Penicillins (ampicillin and ampicillin-clavulanic acid), cephalosporins (cefaclor, cefuroxime, cefixime, ceftriaxone, and cefepime), carbapenems (meropenem and imipenem), macrolides (clarithromycin), tetracylins (tetracycline), fluoroquinolones (levofloxacin and sparfloxacin), amphenicol (chloramphenicol) and sulfonamides (trimethoprim-sulfamethoxazole).

Among the 16 antibiotics assessed in this study, all isolates tested were susceptible to three (Table 2): imipenem (*n* = 700), levofloxacin (*n* = 700) and rifampin (*n* = 104). An additional nine antibiotics had ≤2% of isolates with reduced susceptibility [amoxicillin-clavulanic acid (*n* = 13), cefuroxime (*n* = 14), cefixime (*n* = 2), ceftriaxone (*n* = 1), cefepime (*n* = 3), chloramphenicol (*n* = 2), meropenem (*n* = 3), sparfloxacin (*n* = 3), and tetracycline (*n* = 5)]. Finally, a range of reduced susceptibilities exhibited by the isolates were observed for the remaining four drugs: cefaclor (23.4%), trimethoprim-sulfamethoxazole (29.9%), ampicillin (31.1%), and clarithromycin (46.1%) (Table 2). When comparing NTHi to all typeable isolates combined, the percentage of isolates with reduced susceptibility was higher among NTHi for 10 antibiotics (Table S2).

**TABLE 2:** Antimicrobial susceptibility^*a*^ of invasive H. influenzae isolates, collected through active bacterial core surveillance, United States, 2016 (*n* = 700^*b*^)

Antibiotic	S^*c*^	I	R	NS	RS	MIC range	MIC50	MIC90
Ampicillin	481 (68.7)	22 (3.1)	197 (28.1)	NA	219 (31.3)	≤0.12–>4	0.25	>4
Amoxicillin-Clavulanic Acid	687 (98.1)	NA	13 (1.9)	NA	13 (1.9)	≤2/1–8/4	≤2/1	4/2
Cefaclor^*d*^	536 (76.6)	74 (10.6)	90 (12.9)	NA	164 (23.4)	≤4–>16	≤4	>16
Cefuroxime^*d*^	686 (98.0)	11 (1.6)	3 (0.4)	NA	14 (2.0)	≤0.5–>8	1	4
Cefixime^*d*^	698 (99.7)	NA	n/a	2 (0.3)	2 (0.3)	≤0.12–>1	≤0.12	≤0.12
Ceftriaxone^*d*^	699 (99.9)	NA	n/a	1 (0.1)	1 (0.1)	≤0.03–>2	≤0.03	≤0.03
Cefepime^*d*^	697 (99.6)	NA	n/a	3 (0.4)	3 (0.4)	≤0.12–>2	≤0.12	0.25
Chloramphenicol	698 (99.7)	0 (0.0)	2 (0.3)	NA	2 (0.2)	≤0.5–>4	1	1
Clarithromycin	377 (53.9)	279 (39.9)	44 (6.3)	NA	323 (46.1)	≤0.12–>16	8	16
Imipenem	700 (100.0)	NA	n/a	0 (0.0)	0 (0.0)	≤0.5–4	≤0.5	1
Meropenem	697 (99.6)	NA	n/a	3 (0.4)	3 (0.4)	≤0.06–2	≤0.06	0.12
Levofloxacin	700 (100.0)	NA	n/a	0 (0.0)	0 (0.0)	≤0.03–0.5	≤0.03	≤0.03
Sparfloxacin	697 (99.6)	NA	n/a	3 (0.4)	3 (0.4)	≤0.03–>1	≤0.03	≤0.03
Tetracycline	695 (99.3)	0 (0.0)	5 (0.7)	NA	5 (0.7)	≤0.25–>4	1	1
Trimethoprim-Sulfamethoxazole	491 (70.1)	49 (7.0)	160 (22.9)	NA	209 (29.9)	≤0.06/1.19–>2/38	0.25/4.75	>2/38
Rifampin^*b*^	104 (100.0)	0 (0.0)	0 (0.0)	NA	0 (0.0)	0.25−0.75	0.38	0.5

a Antimicrobial susceptibility was determined for 700 isolates by the broth microdilution method, in accordance with CLSI guideline, for all antibiotics shown except rifampin (39); a subset of the 700 isolates (*n* = 104) were assessed for susceptibility to rifampin using the Etest method.

b Invasive isolates were collected through the Active Bacterial Core Surveillance program, which is active, population- and laboratory-based surveillance and includes 10 catchment areas that covered approximately 44.2 million US residents (13.7% of the population in 2016) (32). During 2016, 767 of 866 confirmed cases (88.6%) included in the ABCs had isolates submitted to CDC; among those, 700 were randomly selected for broth microdilution testing. A subset of the 700 isolates (*n* = 104) were also assessed for rifampin susceptibility by Etest.

c S, susceptible, I, intermediate, R, resistant, NS, nonsusceptible, RS, reduced susceptibility (sum of I, R, and NS), MIC, minimum inhibitory concentration, NA, not applicable (no Clinical Laboratory and Standards Institute [CLSI] breakpoint is defined for the specified susceptibility category).

d Five cephalosporins were included on the broth microdilution panel, including two 2^nd^ generation (cefaclor and cefuroxime), two third generation (cefixime and ceftriaxone) and one fourth generation (cefepime).

For the four antibiotics with >20% of isolates exhibiting reduced susceptibility (ampicillin, cefaclor, clarithromycin, and trimethoprim-sulfamethoxazole), associations with isolate susceptibility and patient age, gender, type of infection, or serotype were assessed. Isolate susceptibility was significantly associated with serotype for each of the four antibiotics tested (*P* ≤ 0.00031 using a corrected alpha level of 0.00313 for significance), but not patient age, gender or the type of infection (Table 3). Reduced susceptibility was more commonly observed in Hie and NTHi isolates: a range of 32.4–70.6% reduced susceptibility was observed for Hie and 25.7–54.1% for NTHi among the four antibiotics. In contrast, reduced susceptibility was less common in the serotypes Hia and Hif: a range of 3.9–15.7% reduced susceptibility was observed for Hia and 11.2–14.6% for Hif. No clear trends were observed for Hib, which is likely due to the small number of isolates in the collection (*n* = 12).

**TABLE 3:** Association of isolate susceptibility to patient age, gender, type of infection or serotype

		Ampicillin, *n*(%^*b*^)			Cefaclor, *n*(%)			Clarithromycin, *n*(%)			Trimethoprim-sulfamethoxazole, *n*(%)		
Isolate susceptibility association	All isolates^*a*^	S^*c*^	RS	*P*-value^*d*^	S	RS	*P*-value	S	RS	*P*-value	S	RS	*P*-value
Age in yrs (*n* = 684^*a*^)													
0 to 5	89	67 (75.3)	22 (24.7)	0.43	72 (80.9)	17 (19.1)	0.65	60 (67.4)	29 (32.6)	0.048	59 (66.3)	30 (33.7)	0.67
6 to 20	26	17 (65.4)	9 (34.6)		19 (73.1)	7 (26.9)		16 (61.5)	10 (38.5)		20 (76.9)	6 (23.1)	
21 to 50	94	67 (71.3)	27 (28.7)		69 (73.4)	25 (26.6)		49 (52.1)	45 (47.9)		64 (68.1)	30 (31.9)	
Over 50	475	319 (67.2)	156 (32.8)		365 (76.8)	110 (23.2)		247 (52.0)	228 (48.0)		337 (70.9)	138 (29.1)	
Gender (*n* = 684^*a*^)													
Female	373	245 (65.7)	128 (34.3)	0.061	282 (75.6)	91 (24.4)	0.44	203 (54.4)	170 (45.6)	0.98	259 (69.4)	114 (30.6)	0.64
Male	311	225 (72.3)	86 (27.7)		243 (78.1)	68 (21.9)		169 (54.3)	142 (45.7)		221 (71.1)	90 (28.9)	
Type of infection (*n* = 602^*a*^)													
Bacteremia without focus	155	104 (67.1)	51 (32.9)	0.56	114 (73.5)	41 (26.5)	0.19	83 (53.5)	72 (46.5)	0.54	103 (66.5)	52 (33.5)	0.53
Meningitis	53	34 (64.2)	19 (35.8)		37 (69.8)	16 (30.2)		32 (60.4)	21 (39.6)		37 (69.8)	16 (30.2)	
Bacteremic pneumonia	394	277 (70.3)	117 (29.7)		311 (78.9)	83 (21.1)		206 (52.3)	188 (47.7)		281 (71.3)	113 (28.7)	
Serotype (*n* = 700^*a*^)													
Hia	51	49 (96.0)	2 (3.9)	<0.00001^*c*^	48 (94.1)	3 (5.9)	*P* < 0.00031^*c*^	43 (84.3)	8 (15.7)	<0.00001^*c*^	45 (88.2)	6 (11.8)	<0.00001^*c*^
Hib	12	7 (58.3)	5 (41.7)		9 (75.0)	3 (25.0)		12 (100.0)	0 (0.0)		3 (25.0)	9 (75.0)	
Hie	34	15 (44.1)	19 (55.9)		20 (58.8)	14 (41.2)		10 (29.4)	24 (70.6)		23 (67.6)	11 (32.4)	
Hif	89	77 (86.5)	12 (13.5)		77 (86.5)	12 (13.5)		76 (85.4)	13 (14.6)		79 (88.8)	10 (11.2)	
NTHi	514	333 (64.8)	181 (35.2)		382 (74.3)	132 (25.7)		236 (45.9)	278 (54.1)		341 (66.3)	173 (33.7)	

a The number of isolates by category varied due to completeness of data. A total of 16 isolates were excluded from age and gender, due to missing data. A total of 98 isolates were excluded from type of infection for missing data or a clinical presentation other than bacteremic pneumonia, bacteremia without focus, or meningitis. No isolates were excluded for serotype.

b Percentages may not add up to 100, due to rounding.

c S, susceptible; RS, reduced susceptible; Hia, H. influenzae serotype a; Hib, H. influenzae serotype b; Hie, H. influenzae serotype e; Hif, H. influenzae serotype f; NTHi, nontypeable H. influenzae.

d Chi square goodness of fit tests were conducted with a significance of *P* < 0.05. Because 16 chi square tests were performed, a Bonferroni correction was applied; a corrected alpha level of 0.00313 was used for significance. No significant values were identified for age, gender, or type of infection.

Isolates exhibited the highest proportion of reduced susceptibility to ampicillin among clinically relevant antibiotics, with 3.1% ampicillin-intermediate (MICs = 2 μg/mL) and 28.1% ampicillin-resistant (MICs = 4 or >4 μg/mL) isolates detected (Table 2). Among the ampicillin-resistant isolates, 175 (88.8%) contained a β-lactamase gene (*bla_ROB-1_ n* = 8 and *bla_TEM-1_ n* = 167); however, eight ampicillin-susceptible isolates with a *bla_TEM-1_* gene were also detected, each containing the *bla_TEM-1_* reference sequence. Among the 22 ampicillin-resistant isolates without a β-lactamase gene (BLNAR isolates), 13 (59.0%) had characterized *ftsI* mutations: seven belonged to group I (N526K); 1 belonged to group II (R517H); 4 belonged to group III (N526K, S385T, L389F); and 1 belonged to group III-like (R517H, S385T, L389F). Nine ampicillin-resistant isolates had no known mechanism of resistance. All 22 (100%) ampicillin-intermediate isolates also had characterized *ftsI* mutations (group I *n* = 21 and group III *n* = 1) and were negative for both β-lactamase genes.

Reduced susceptibility to the amoxicillin-clavulanate, which contains a β-lactamase inhibitor, was less common: 13 (1.9%) amoxicillin-clavulanate-resistant (MICs = 8/4 μg/mL) isolates were observed. Among the 13 amoxicillin-clavulanate-resistant isolates, all had reduced susceptibility to ampicillin (12 ampicillin-resistant and 1 ampicillin-intermediate) and 12 (92.3%) had characterized *ftsI* mutations (group I *n* = 7; group III *n* = 5). The proportion of amoxicillin-clavulanate-resistant isolates (1.9%) was comparable to the proportion of BLNAR isolates (3.1%) in the collection; among the 22 BLNAR isolates, 72.7% (*n* = 16) isolates were amoxicillin-clavulanate-susceptible and 23.7% (*n* = 6) were amoxicillin-clavulanate-resistant.

The number of isolates exhibiting reduced susceptibility to cephalosporins varied by antibiotic generation. Reduced susceptibility to second generation drugs was more common; 10.6% cefaclor-intermediate (MICs = 16 μg/mL) and 12.9% cefaclor-resistant (MICs > 16 μg/mL) as well as 1.6% cefuroxime-intermediate (MICs = 8 μg/mL) and 0.4% (MICs > 8 μg/mL) cefuroxime-resistant isolates observed. Among these, 67.7% (*n* = 111) of isolates with reduced susceptibility to cefaclor had a β-lactamase and 2.1% (*n* = 3) of isolates with reduced susceptibility to cefuroxime had a β-lactamase. In contrast, reduced susceptibility to 3rd and 4th generation cephalosporins was rare. Among the 3rd generation drugs, two (0.3%) cefixime-nonsusceptible isolates were observed (MIC >1 μg/mL) and both were β-lactamase negative; one was also cefaclor-intermediate and contained a group I *ftsI* mutation, while the second isolate was resistant to four other β-lactam agents (ampicillin, ampicillin-sulbactam, cefaclor and cefuroxime) and contained group III *ftsI* mutations. The only ceftriaxone-nonsusceptible isolate (MIC >2 μg/mL) was also cefaclor-intermediate, contained the group I *ftsI* mutation, and was β-lactamase negative. Finally, three nonsusceptible isolates to the only 4th generation cephalosporin, cefepime (MICs > 2 μg/mL), were observed. However, all three were susceptible to all other β-lactam antibiotics and did not have any characterized mutations in the *ftsI* gene; a β-lactamase gene was present in one of the three isolates.

## DISCUSSION

Investigating antimicrobial susceptibility and potential mechanisms of resistance are important for ensuring that current antibiotic treatment and chemoprophylaxis recommendations remain effective. Here, we assessed invasive H. influenzae isolates, collected through population-based surveillance in 2016, for susceptibility to 16 antibiotics and demonstrated that reduced susceptibility to clinically relevant drugs remained uncommon, except for ampicillin. Genetic analysis confirmed that ampicillin resistance was predominantly associated with the presence of a β-lactamase gene. Approximately a quarter of the isolates were susceptible to all 16 antibiotics and another third had reduced susceptibility to only one antibiotic class. High levels of reduced susceptibility (>20% of all isolates tested) were observed for only four drugs (ampicillin, cefaclor, clarithromycin and trimethoprim-sulfamethoxazole) and susceptibility status was associated with isolate serotype but not patient age, gender, or type of infection.

The susceptibility patterns observed within the 2016 invasive H. influenzae isolates were consistent with previous reports, indicating that trends have been relatively stable in the United States. Either low levels or a complete absence of reduced susceptibility have been reported for rifampin, amoxicillin-clavulanate, and third or fourth generation cephalosporins, which was also observed in this analysis (10–17). In contrast, the proportion of reduced susceptibilities to ampicillin, cefaclor, clarithromycin, and trimethoprim-sulfamethoxazole have been more commonly reported, albeit with some variation among studies, and were also observed in this survey (11–15). In addition, this analysis confirmed that ampicillin resistance is continuing to remain relatively stable since 2003 within the population (13); resistance was detected in approximately a quarter of isolates and predominantly associated with the presence of the *bla_TEM-1_* gene, while BLNAR isolates, containing *ftsI* mutations, remained uncommon (5). It has been reported that imipenem resistance has been reported in BLNAR isolates (26); however, no reduced susceptibility to imipenem was observed in this study.

Overall, detection of genetic resistance mechanisms supported the phenotypic susceptibility testing results and strengthened the findings of this survey; however, a few exceptions were observed. First, we identified isolates with resistance mechanisms that were phenotypically susceptible. For example, eight ampicillin-susceptible strains with a full-length *bla_TEM-1_* gene were detected; phenotypic nitrocefinase testing (data not shown) showed that only 3 of these 8 strains were positive for β-lactamase activity (observed ampicillin MICs were ≤0.12, 0.5, and 1 μg/mL). Additional characterization by Etest for susceptibility testing and genomics investigations into the *bla_TEM-1_* gene insertion site could help identify the cause of these discrepancies. Second, we observed isolates with reduced susceptibility that lacked a known resistance mechanism; this observation could be caused by novel resistance mechanisms or a potential misidentification of the reduced susceptibility phenotype. The most notable examples from this analysis were the three cefepime nonsusceptible isolates. Because these isolates were also fully susceptible to all of the earlier generation cephalosporins on the panel, it is most likely that the growth observed by broth microdilution is related to spheroblast production, which can result in inappropriately high MICs for β-lactam agents (27, 28). In addition, nine BLNAR isolates without well characterized *ftsI* mutations were identified; better characterization of all possible *ftsI* mutations affecting susceptibility to penicillins will be important to improve our understanding of mechanisms contributing to BLNAR phenotypes. Multifactorial genetic mechanisms associated with imipenem resistance, mutations in PBP3 and the AcrR regulator, could not be evaluated because no reduced susceptibility was observed (9, 29, 30); the mechanism is too poorly characterized for interpretation of any observed PBP3 and AcrR mutations in susceptible isolates.

This study was subject to several limitations. First, the presence of β-lactamase genes was assessed using a genomics screen, not a phenotypic nitrocefinase test; to resolve potential discrepancies between susceptibility and the detection of β-lactamase genes in future studies, both methods could be used. Second, most drugs on the antibiotic panel contained a range of dilutions that were only within the susceptible or intermediate ranges for that antibiotic, making identification of the MIC within the nonsusceptible or resistant range not possible; thus, specific patterns or trends of MICs within the nonsusceptible or resistant ranges could not be assessed. In future analyses, use of MIC panels that include dilution ranges that encompass all possible interpretation categories for each drug may improve our understanding of the observed nonsusceptible or resistant phenotypes; these assessments could also help gain additional insight into the epidemiological cutoff values (ECOFFs). Third, specifically for the β-lactam agents, we observed a trailing growth phenotype that made MIC identification challenging. Previous studies have shown that some H. influenzae strains produce spheroblasts, which result in inappropriately high MICs for β-lactam agents (27, 28). The trailing growth was observed as a nearly translucent pellet within the HTM broth and most prominent in typeable isolates; thus, it is possible this observed phenotype was related to potential spheroblast production. To ensure accurate MIC identification in this study, all isolates exhibiting the trailing growth phenotype were repeated for confirmation; however, in the future, it may be helpful to explore additional media types for H. influenzae broth microdilution, such as Mueller-Hinton-Fastidious broth, which is used in the European Committee on Antimicrobial Susceptibility Testing (EUCAST) method (31). Fourth, the broth microdilution method used in this study may not detect imipenem heteroresistance phenotypes as well as agar-based methods and has been described previously (9, 26, 29). Finally, the genetic basis of resistance in H. influenzae is poorly defined and the H. influenzae genome is highly variable, making the association of observed genetic mutations and susceptibility phenotypes more challenging. It will be important for novel genetic studies to determine if there is a causal relationship with susceptibility.

Overall, this 2016 antimicrobial resistance survey of invasive H. influenzae isolates demonstrated that, with the exception of ampicillin, the US H. influenzae population remained susceptible to clinically relevant antibiotics. Detection of approximately a quarter ampicillin-resistant and β-lactamase containing strains demonstrates that resistance mechanisms can be acquired and sustained within the H. influenzae population, highlighting the continued importance of antimicrobial resistance surveillance for H. influenzae to monitor susceptibility trends and mechanisms of resistance.

## MATERIALS AND METHODS

### Case definition and bacterial isolate collection.

Surveillance for H. influenzae was conducted as part of Active Bacterial Core Surveillance (ABCs). ABCs is an active, population- and laboratory-based surveillance system for invasive bacterial pathogens of public health importance and the surveillance area includes 10 sites that covered approximately 44.2 million US residents in 2016 (13.7% of the population) (32). For invasive H. influenzae disease, a confirmed case is defined as isolation of H. influenzae by culture or detection of H. influenzae by PCR from a normally sterile body site Council of State and Territorial Epidemiologists (CSTE) case. Epidemiologic information, including patient age, gender, and type of infection, was abstracted from medical records. Cases of invasive H. influenzae disease were categorized by the type of infections using the following criteria: (i) meningitis if a clinical diagnosis of meningitis was recorded in the medical record and H. influenzae was isolated from CSF or other sterile sites; (ii) bacteremic pneumonia if pneumonia was recorded in the patient’s medical record and H. influenzae was isolated from blood or pleural fluid; and (iii) bacteremia without focus if H. influenzae was isolated from blood with no localized clinical syndrome.

Invasive H. influenzae isolates, collected during 2016, were submitted to CDC through ABCs. During 2016, 767 of 866 confirmed cases (88.6%) reported from ABCs sites had isolates submitted to CDC [CDC unpublished]; among those, 700 were randomly selected and de-identified for inclusion in this antimicrobial susceptibility survey. This activity was reviewed by CDC and determined to be public health evaluation; patient consent and institutional review board review were not required.

### Laboratory characterization of H. influenzae isolates.

H. influenzae species and serotype were confirmed by real-time-PCR and slide agglutination (33–35). Isolate genomes were sequenced using an Illumina MiSeq and multilocus sequence typing (MLST) was performed as described (36). Resistance mechanisms related to clinically relevant antibiotic classes, including ampicillin, amoxicillin-clavulanate, and the cephalosporins, were investigated. Genes were identified by a BLAST search of genome assemblies using NCBI reference sequences for H. influenzae *bla_ROB-1_* (NC_019178), and the PubMLST full-length sequences for *ftsI* (HAEM1263) and *bla_TEM-1_* (HAEM0118) genes (37). Amino-acid mutations in *ftsI* were identified by aligning the translated sequence to allele 1 from PubMLST using BioPython (38). Genome assemblies were submitted to PubMLST. All raw read data have been deposited in the NCBI Sequence Read Archive (SRA) under BioProject accession number PRJNA815855.

### Antimicrobial susceptibility.

H. influenzae isolates were tested by broth microdilution, in accordance with the Clinical and Laboratory Standards Institute (CLSI) M100, 30^th^ Edition (39). The Haemophilus and Streptococcus (HPB1) lyophilized antibiotic panel (Thermo Scientific Sensititre) was used and contained the following antimicrobial dilution series (μg/mL): ampicillin (0.12–4), ampicillin-sulbactam (1/0.5–2/1), amoxicillin-clavulanic acid (2/1–16/8), cefaclor (4–16), cefuroxime (0.5–8), ceftriaxone (0.03–2), cefixime (0.12–1), cefepime (0.12–2), chloramphenicol (0.5–4), clarithromycin (0.12–16), imipenem (0.5–4), meropenem (0.06–2), levofloxacin (0.03–4), sparfloxacin (0.03–1), tetracycline (0.25–4), and trimethoprim-sulfamethoxazole (0.06/1.19–2/38). Any isolate exhibiting a trailing growth phenotype by broth microdilution was repeated for confirmation (additional information about the observed trailing growth is in the discussion limitations). Ampicillin-sulbactam was present on the panel (1/0.5 and 2/1 μg/mL) but was excluded from this analysis due to poor reproducibility among broth microdilution replicates and only two dilutions being available on the panel (data not shown). Three quality control strains, H. influenzae ATCC 49766 and ATCC 49247, and Streptococcus pneumoniae ATCC 49619 were also included during testing. MICs were interpreted as susceptible, intermediate, resistant, or nonsusceptible based on the established CLSI breakpoints (39). Isolates were defined as having reduced susceptibility if they were intermediate, resistant or nonsusceptible.

Because rifampin was not present on the lyophilized panel, 104 isolates were selected to also be assessed by Etest, using the Rifampicin RI Etest strip (bioMérieux). Both typeable (Hia *n* = 29, Hib = 3, Hie = 16, Hif = 5) and NTHi (*n* = 51) isolates were included; more than half (29/51) of the available Hia isolates were included because of the recent updated recommendations for use of rifampin for Hia disease prophylaxis (2). Susceptibility testing was performed according to manufacturer specifications, including use of the S. pneumoniae ATCC 49619 control strain during testing. MICs were interpreted as susceptible, intermediate, or resistant based on the established CLSI breakpoints (39).

Antibiotic classes were defined as the following: Penicillins (ampicillin and amoxicillin-clavulanic acid), cephalosporins (cefaclor, cefuroxime, cefixime, ceftriaxone, and cefepime), carbapenems (meropenem and imipenem), macrolides (clarithromycin), tetracylins (tetracycline), fluoroquinolones (levofloxacin and sparfloxacin), amphenicol (chloramphenicol) and sulfonamides (trimethoprim-sulfamethoxazole).

### Statistical analysis.

To assess possible associations between isolate susceptibility and patient age, gender, type of infection, or serotype, isolates were categorized as either susceptible or having reduced susceptibility. Patient ages were grouped as 0–5 years, 6–20 years, 21–50 years, and over 50 years. For this analysis, only the four antibiotics with >20% of isolates with reduced susceptibility were included (ampicillin, cefaclor, clarithromycin and trimethoprim-sulfamethoxazole), each representing a unique antibiotic class. Sixteen isolates were excluded from the age and gender analyses, due to missing data; 98 isolates were excluded from the type of infection analysis, due to missing data or a clinical presentation other than bacteremic pneumonia, bacteremia without focus, or meningitis. Data were imported into R (version 4.0.3). Chi square goodness of fit tests were conducted with a significance of *P* < 0.05 and a Bonferroni correction was applied; a corrected alpha level of 0.00313 was used for significance.
